# Preoperative imaging contributes to pathologically complete resection of the urachal remnant by determining an appropriate surgical approach without unnecessary and excessive surgical invasion: a retrospective study

**DOI:** 10.1186/s12894-022-01153-x

**Published:** 2022-12-19

**Authors:** Yoichi Nakagawa, Wataru Sumida, Hizuru Amano, Hiroo Uchida, Akinari Hinoki, Chiyoe Shirota, Satoshi Makita, Masamune Okamoto, Seiya Ogata, Aitaro Takimoto, Shunya Takada, Daiki Kato, Yousuke Gohda

**Affiliations:** 1 Department of Pediatric Surgery, Nagoya University Graduate School of Medicine, 65 Tsurumai-cho, Showa-ku, Nagoya, 466-8550 Japan; 2 Department of Rare/Intractable Cancer Analysis Research, Nagoya University Graduate School of Medicine, 65 Tsurumai-cho, Showa-ku, Nagoya, 466-8550 Japan

**Keywords:** Urachal remnant, Trans-umbilical approach, Laparoscopic-assisted trans-umbilical approach, Pediatrics

## Abstract

**Background:**

The urachus is an embryonic structure that connects the bladder to the allantois during early embryonic development. Occasionally, it fails to disappear at birth, leading to a case of urachal remnant (UR). This study aimed to determine whether our policy for selecting an appropriate UR resection approach is valid. We performed preoperative imaging to examine whether UR continued toward the bladder apex. If so, the UR and bladder apex were excised using the trans-umbilical approach, in addition to laparoscopy, if necessary. If preoperative imaging indicated that the UR ended near the umbilicus, the UR from the umbilicus to the duct end was resected. Pathological evaluations were performed to determine the appropriateness of the surgical approach indicated by preoperative imaging.

**Methods:**

We retrospectively reviewed pediatric patients with UR who underwent surgery between 2015 and 2021. Their background characteristics and surgical outcomes were evaluated.

**Results:**

Twenty patients with UR were included (median age, 7 [interquartile range, 2–10.25] years). UR continued toward the bladder apex in 10 patients and ended near the umbilicus in 10 patients. Urachus tissue at the bladder site was observed when the UR and bladder apex were excised. When UR was resected from the umbilicus to the duct end, urachus tissue was not pathologically detected at the resection margin.

**Conclusion:**

Our policy results in complete resection without excessive surgical invasion.

## Background

The urachus is an embryonic structure that connects the bladder to the allantois during early embryonic development. Occasionally, it fails to disappear at birth, leading to a case of urachal remnant (UR), which is observed in up to 32% of all bladder examinations of adults aged > 32 years [1]. UR treatment comprises conservative therapy and surgery. Historically, UR resection has been recommended to prevent urachal carcinomas; however, a recent study proposed that 5721 urachal remnants would need to be excised to prevent urachal adenocarcinoma [2]. For symptomatic cases, surgical resection is an appropriate treatment option; however, excision of the bladder apex is controversial in some cases.

At our institution, surgical intervention is performed for symptomatic UR. Our policy regarding UR involves preoperative imaging to assess whether UR continues toward the bladder to ensure that complete resection can be performed and pathologically confirmed. If UR does not continue to the bladder apex, that is, in case of urachal sinus, the trans-umbilicus (TU) approach is selected to excise UR because it is enough to completely excise the urachal sinus. However, if UR continues to the bladder apex, that is, in case of a patent urachus, the TU approach is considered as the first option, and laparoscopy is added (laparoscopy-assisted trans-umbilicus [LATU] approach) if the bladder apex cannot be removed from the umbilical site by the TU approach. If preoperative imaging shows that the UR does not continue to the bladder apex and is limited near the umbilicus, and intraoperative findings are consistent with preoperative findings, UR is resected at the fibromuscular cord-like structure, and the bladder apex is not excised. If the UR continues to the bladder apex, the UR and bladder apex are both excised.

Several studies have evaluated the operative time, cosmetic appearance, and surgical outcomes of the TU and LATU approaches for UR excision [3–5]. However, no studies have discussed whether an appropriate approach to UR excision can be determined using preoperative imaging and confirmed by pathological findings. This study aimed to evaluate pathological examination results to assess the validity of our policy of performing preoperative imaging to determine an appropriate UR resection approach.

## Methods

We retrospectively reviewed pediatric patients with UR who underwent surgery at our department between 2015 and 2021. We collected background characteristics, preoperative imaging results, surgical procedures, pathological examination results, and surgical outcomes from medical records.

### Diagnosis

We performed preoperative imaging, including ultrasonography (US), computed tomography (CT), and magnetic resonance imaging (MRI), in all UR cases and preoperatively determined whether UR continued toward the bladder apex. Upon admission, all patients complained of symptoms, including abdominal pain, exudates, and hematuria. At our institution, we screened some diseases by US and confirmed a diagnosis of UR at initial visit. In patients referred from other hospitals, they were already diagnosed with UR by US, CT, or MRI. In these patients, some were suspected of UR and subsequently diagnosed; however, others were incidentally diagnosed with UR by CT or MRI. In principle, US was the main diagnostic tool. When US was unable to distinguish whether UR was in the urachal sinus or patent urachus, MRI was performed to clearly diagnose UR type. CT was not basically performed; however, it was performed when patients with UR were suspected of malignant tumors. Nevertheless, most cases already underwent CT or MRI before referral visit because our institution is a tertiary hospital.

### Surgical policy

**Fig. 1:**
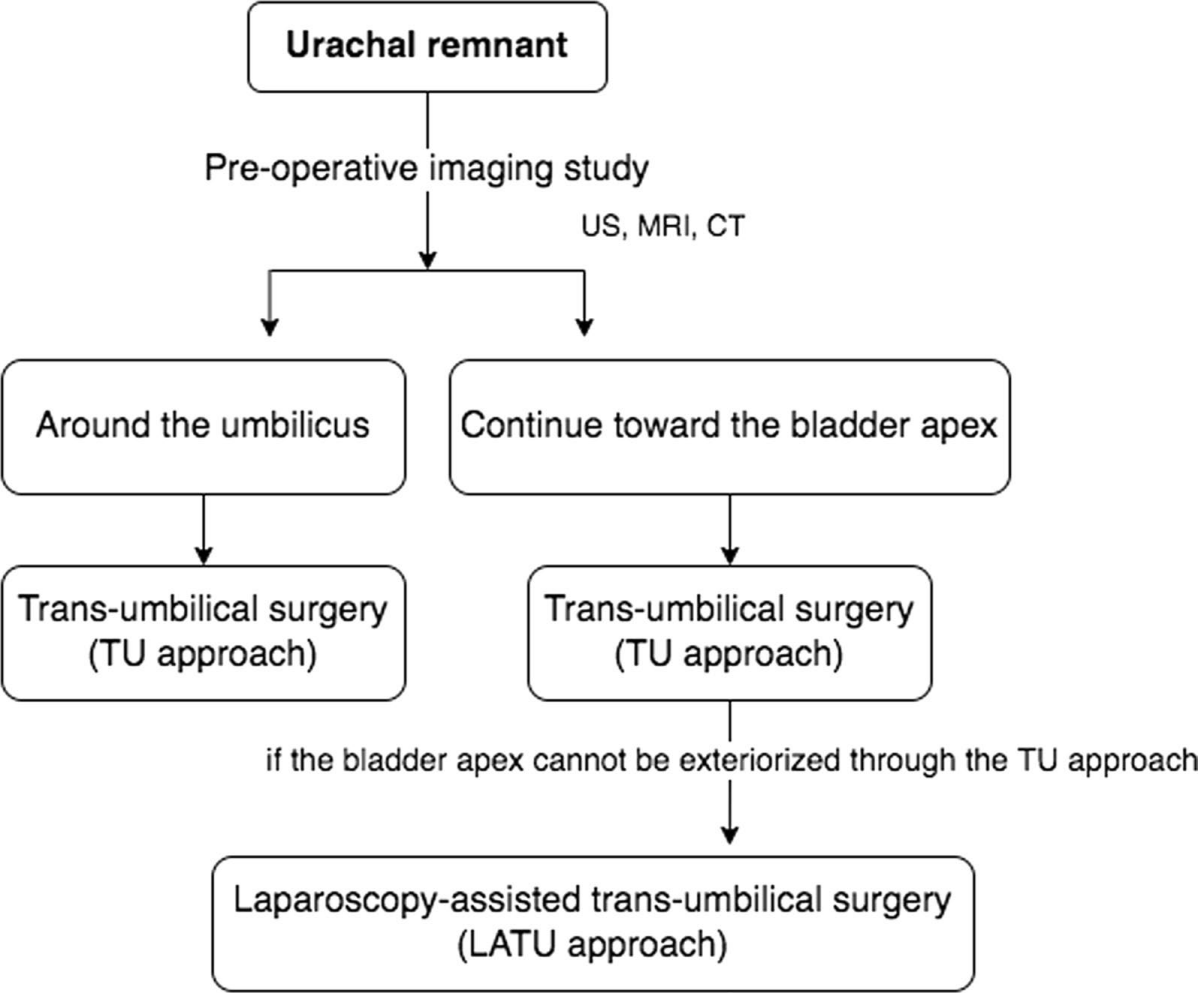
Flowchart showing how the surgical approach was selected for urachal remnant

When it was preoperatively determined that UR was limited near the umbilicus, the TU approach was selected. When it was preoperatively determined that UR continued toward the bladder apex, the TU approach was initially selected. However, the LATU approach was added when we were unable to remove the bladder apex from the umbilical site. In our experience, the TU approach was usually unable to completely excise the UR, including the bladder apex, in old patients. In these cases, laparoscopy was added, and the LATU approach was performed (Fig. 1).

**Fig. 2:**
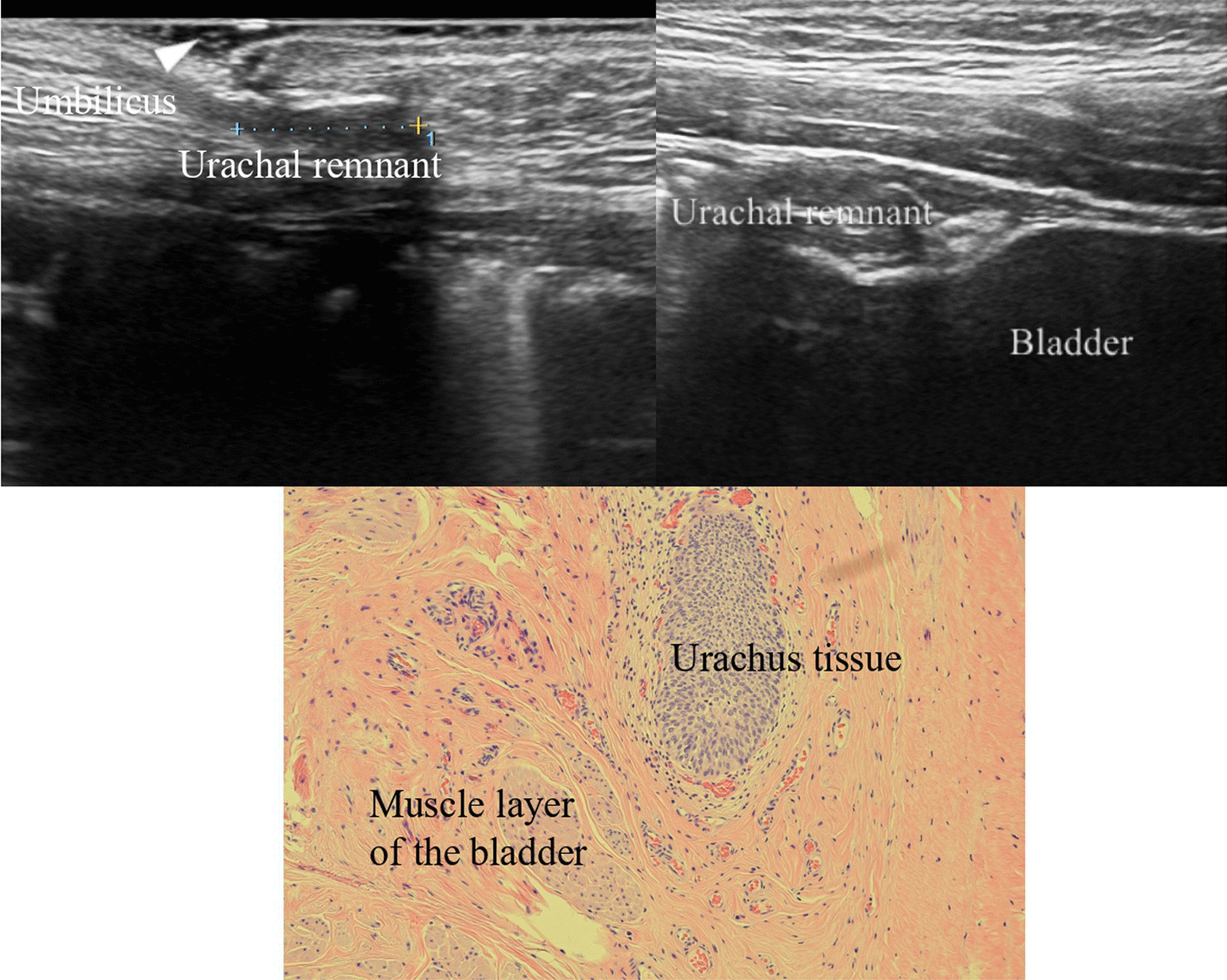
Ultrasonography shows that the urachal remnant continued toward the bladder apex. Pathological findings indicate urachus tissue at the bladder apex

**Fig. 3:**
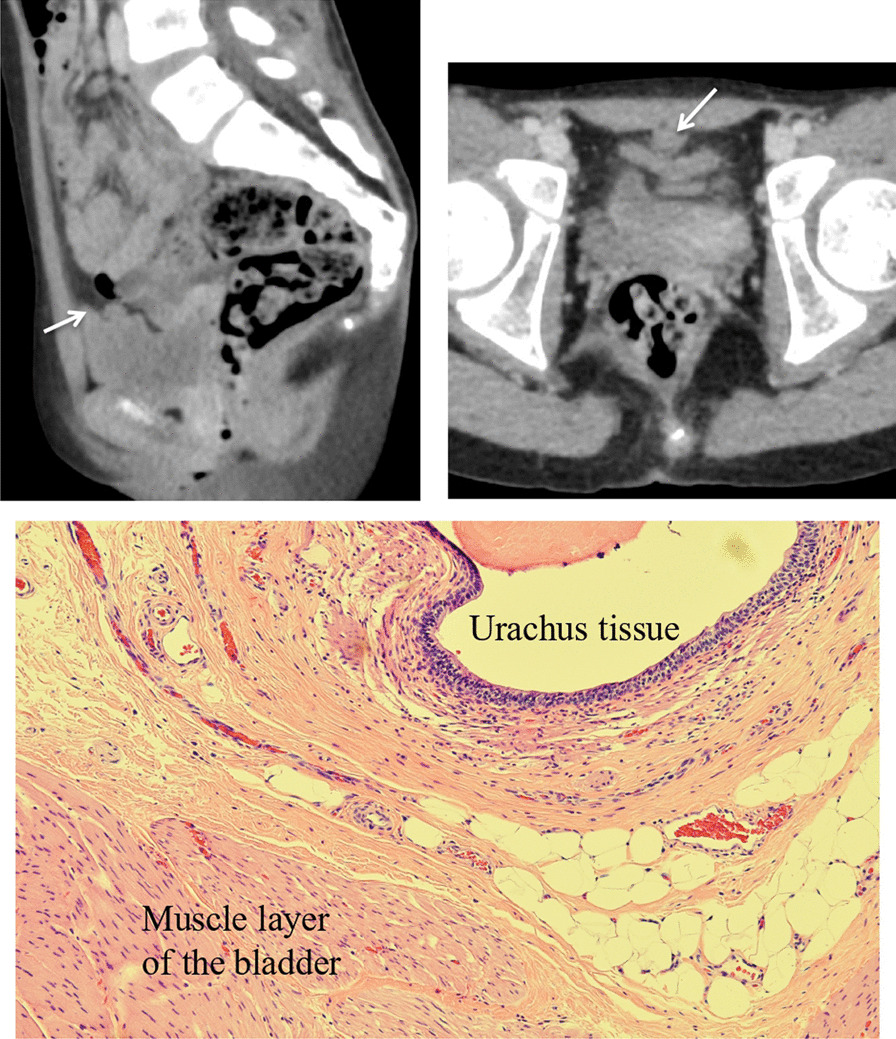
CT showed the urachal remnant at the bladder apex (white arrow). Pathological findings indicated urachus tissue at the bladder apex

**Fig. 4:**
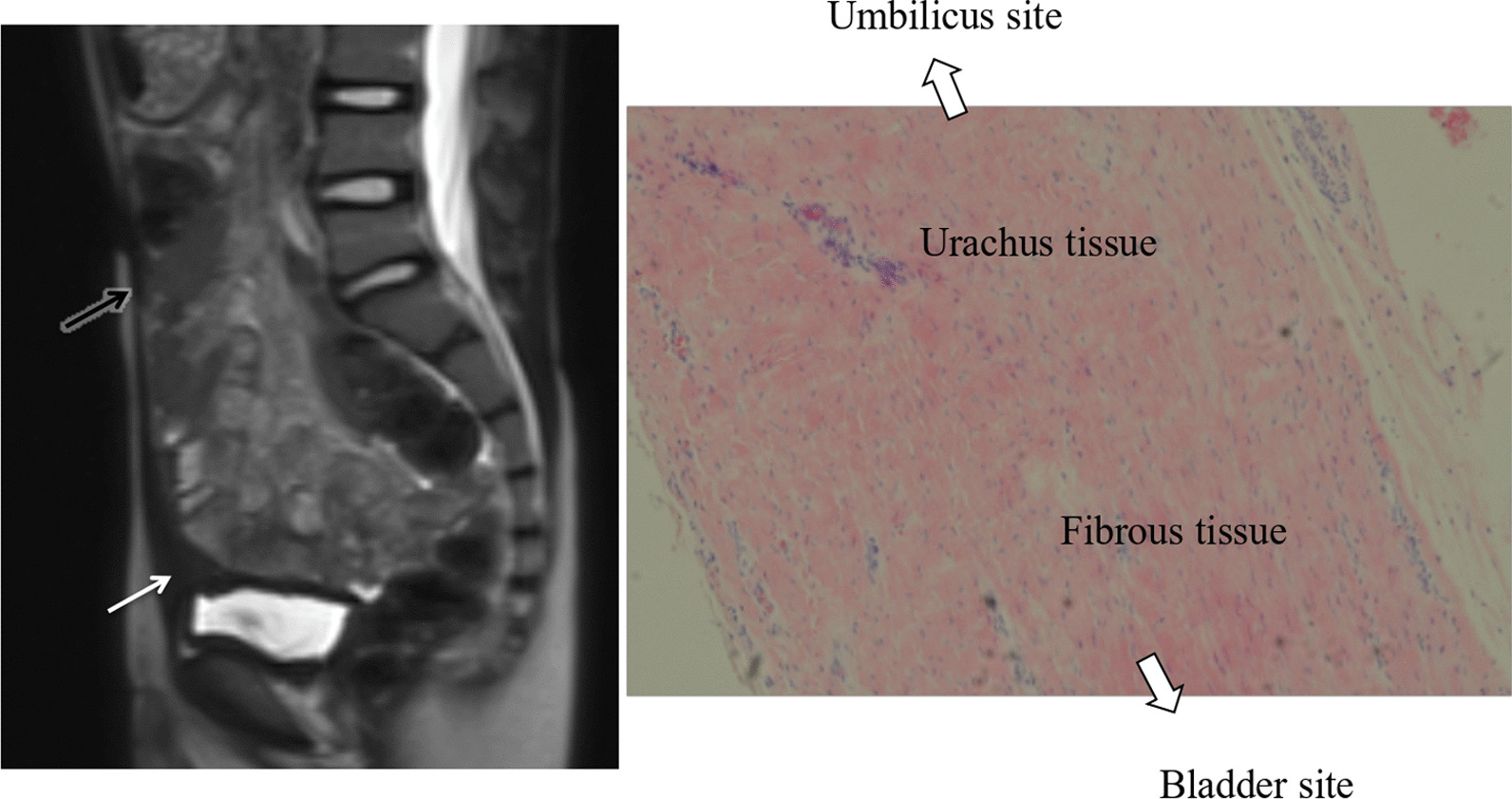
MRI (T2-weighted) shows that there is no urachal remnant at the bladder apex (white arrow). The urachal remnant was resected at the fibromuscular cord-like structure (black arrow). Pathological findings indicated urachus tissue at the umbilicus site and no urachus tissue at the fibromuscular cord-like structure; only fibrous tissue was observed

### Surgical procedure: TU approach

The patient was placed in the supine position with general anesthesia being administered by tracheal intubation. Subsequently, a urethral catheter was placed in the bladder. A circumumbilical incision (subumbilical arch) was performed, and the linea alba was cut caudally from the umbilicus. The urachus (median umbilical ligament) located in the preperitoneal fat was identified and traced to the bladder apex to some extent without opening the abdominal cavity. The umbilicus skin attached to the urachus was removed. When it was preoperatively determined that UR did not continue to the bladder apex, the end of UR was identified intraoperatively and completely resected. When it was preoperatively determined that the UR continued to the bladder apex, the UR was traced to the bladder apex, if possible. If the bladder apex could not be removed using the TU approach, the LATU approach was performed.

### Surgical procedure: LATU approach

When the UR preoperatively continued to the bladder apex, and the bladder apex could not be removed using the TU approach, the LATU approach was added. The latter surgical approach was performed similarly to the former approach. After tracing UR, Alexis^®^ wound protector retractors (size XS; Applied Medical Resources Corporation, Rancho Santa Margarita, CA, USA) were inserted into the abdominal cavity through the umbilicus incision, and FREE ACCESS (XS-size; TOP Corporation, Tokyo, Japan) with two 5-mm trocars was attached to allow camera and walking ports. Another 5-mm trocar was inserted at the left lateral abdominal wall to allow a working port. Pneumoperitoneum was maintained by an insufflation pressure of 8 mmHg. Using laparoscopy, the urachus was identified and dissected stepwise toward the bladder apex. The urachus was completely extracted along with the bladder muscular layer with or without perforation of the bladder membrane. The resected site of the bladder muscle was ligated with 5 − 0 absorbable sutures or Endoloop PDS II (Ethicon Inc., Cincinnati, OH, USA). Subsequently, the bladder was filled with saline solution using the urethral catheter to check for any perforations.

### Postoperative course

Three hours after recovering from anesthesia, oral intake was allowed. After resection of the bladder apex, a urethral catheter was placed for 2 days and removed on postoperative day 2. If resection of the bladder apex was not performed, the urethral catheter was subsequently removed on mobilization day.

### Statistical analysis

Continuous variables are expressed as the median and interquartile range. The Wilcoxon Signed Rank and Fisher’s exact tests were performed to compare continuous and discrete variables, respectively. A *p*-value < 0.05 was considered statistically significant. All statistical analyses were performed using R software 4.1.2 [6]. We evaluated whether the policy of our institution to determine the surgical approach based on preoperative imaging was appropriate.

### Ethical approval

This study was performed in accordance with the ethical standards indicated in the 2000 Declaration of Helsinki and its later amendments or comparable ethical standards. Written informed consent was obtained from the legal guardians of the patients. This study was approved by the institutional review board of the Nagoya University Graduate School of Medicine (approval number: 2021–0395).

## Results

Twenty patients with UR were included in this study. All patients had symptoms such as abdominal pain, exudates, and infection. The median patient age was 7 (interquartile range, 2–10.25) years. Boys comprised 75% (15/20) of patients. Preoperative imaging was performed, and all 20 patients underwent US and were diagnosed with UR. Five cases of UR were diagnosed by US only. MRI was taken in six cases; three cases were evaluated with MRI because US could not determine whether UR continued toward the bladder apex, and the other three cases already underwent MRI before being referred, even though these cases were possibly diagnosed by US only. CT was performed in nine cases; one was evaluated with CT due to suspicions of a malignant bladder tumor, another one due to repeated abscess formation, and the other seven cases did not require CT evaluation, but it had already been performed at another hospital before referral to our institutions.

Ten cases of UR were limited near the umbilicus by pre- and intra-operative findings (Table 1); therefore, UR was excised at the fibromuscular cord-like structure using the TU approach. Moreover, UR was found to have continued toward the bladder apex by pre- and intraoperative findings in 10 cases (Table 2), all of which initially underwent the TU approach. Seven of the 10 UR cases required laparoscopy and underwent the LATU approach. The TU approach was able to excise UR completely for patients aged less than two years.

No early or long-term postoperative complications occurred. In UR cases limited at near the umbilicus as determined by pre- and intraoperative findings, all 10 cases were successfully excised by the TU approach. In cases of UR that continued toward the bladder apex, both the LATU and TU approaches were performed. The LATU approach involved a longer operative time than the TU approach (LATU: median, 131 [interquartile range, 106.5–140.5] min vs. TU: median, 59 [interquartile range, 56–70] min; *p* = 0.02). The median hospitalization and urinary catheter placement period differed between the UR limited at near the umbilicus and UR continued toward the bladder apex, but this was due to differences in the postoperative management course.

Pathological findings indicated that the urachus tissue at the bladder site was present when preoperative imaging showed that the UR continued toward the bladder apex. When preoperative imaging showed that the UR did not continue to the bladder apex, urachus tissue was present near the umbilicus but ended at the fibromuscular cord-like structure and did not exist at the resected margin. A pathological examination of the resected sites (i.e., fibromuscular cord-like structure) revealed no urachus tissue; however, urachus tissue was pathologically observed near the umbilicus. All cases of UR that did not continue to the bladder apex were appropriately resected with negative pathological margins (Table 3).

Some imaging results and pathological findings are shown in Figs. 2, 3 and 4. Figure 2 shows a 6-year-old boy whose preoperative US evaluation indicated that the UR continued toward the bladder apex. The TU approach was initially performed; however, the LATU approach was required to excise the UR and bladder apex in the end. Pathological findings revealed the existence of urachus tissue at the bladder apex. Figure 3 shows an 11-year-old girl whose preoperative CT evaluation indicated that UR existed at the bladder apex. The same operative process was performed. Pathological findings revealed the existence of urachus tissue at the bladder apex. Figure 4 shows a 10-year-old boy whose preoperative MRI evaluation indicated that the UR did not continue toward the bladder apex. The TU approach was sufficient for the complete excision of the UR, and the UR was resected at the fibromuscular cord-like structure. Pathological findings revealed fibrous tissue but no urachus tissue at the resected margin.

## Discussion

During this study, preoperative imaging findings indicating whether UR continued toward the bladder apex were consistent with the positive pathological findings at the bladder site. However, when preoperative imaging showed that the UR was near the umbilicus and did not continue toward the bladder apex, both intraoperative findings and pathological examination results revealed fibromuscular tissue only. There was no urachus tissue at the resected site, and the UR ended near the umbilicus. All cases of UR were appropriately resected, and there was a negative pathological remnant. Our institution’s policy (i.e., obtaining preoperative imaging findings and intraoperative findings to determine whether bladder apex removal is necessary) was found to be acceptable.

UR cases usually present with lower abdominal pain and infection near the umbilicus. For pediatric cases, the differential diagnosis of these symptoms includes omphalitis, omphalomesenteric duct, and umbilical granuloma. UR at the umbilical pilonidal sinus is also a possibility for adult cases. Imaging methods such as US are useful for diagnosis; however, a definitive diagnosis confirmed by pathology is essential. There are four types of UR: patent urachus, umbilical-urachal sinus, urachal cyst, and vesico-urachal diverticulum URs [7]. Nine of the 20 (45%) patients in our study had an patent urachus that continued toward the bladder apex.

The treatment strategy for UR is controversial. The complete resection of UR has been historically performed owing to the risk of urachal carcinoma for both adults and children attributable to epithelial or mesenchymal urachal neoplasms that can occur with UR [8, 9]. Malignant urachal neoplasms are usually low-grade and diagnosed at advanced stages [10]. Therefore, complete excision of an asymptomatic UR, even in pediatric patients, has been performed to prevent future malignant neoplasms. However, a recent study showed that only one out of every 5721 cases of UR develop into urachal adenocarcinoma [2]. Another study reported that conservative follow-up is an option for patients with UR younger than 1 year [11, 12] and for those undergoing initial drainage treatment [13]. Each individual pediatric patient with UR should be treated based on the imaging results. In some cases, simple and asymptomatic lesions are not excised; however, in other cases, large and suspicious lesions are excised [2]. We treated cases of symptomatic UR; however, we did not consider the prophylactic excision of asymptomatic UR to be necessary for children.

There are two approaches to UR excision: TU and LATU. The laparoscopic excision of UR has been first reported in 1992 [14]. Several modified laparoscopic surgery techniques for UR have been developed and reported, with similar surgical outcomes and good cosmetic appearance [3, 4]. There are several laparoscopic UR excision methods in terms of port placement, arrangements, and bladder suturing; however, we adopted the LATU approach for excising UR. We considered the preoperative imaging of UR to be appropriate for determining the surgical approach and margin required. Compared to the LATU approach, the TU approach has the advantage of being able to be performed in the preperitoneal space without opening the abdominal cavity to prevent late complications of postoperative obstruction. In our experience, for children younger than two years, the TU approach is sufficient for the complete excision of UR, including the bladder apex, without opening the abdominal cavity. However, in some cases, the TU approach is difficult to completely excise the UR, including the bladder apex, without adding the Y-shape midline skin incision. Cosmetic appearance is an important factor for pediatric patients with UR; therefore, we added a laparoscopic procedure, that is, the LATU approach, in patients with UR unable to be excised completely by the TU approach.

US, CT, and MRI are useful for diagnosing UR [15, 16]. We performed preoperative imaging for all patients. US is the first choice for diagnosing UR at our institution and was performed for all patients. We consider US to be sufficient for diagnosing UR. Because of the risk of radiation exposure, other imaging methods, especially CT, should be adopted only for cases suspected with malignant tumors. MRI does not expose patients to radiation; however, obtaining images with MRI is time-consuming and often requires sedation in young children. It is easier to perform imaging examinations when the bladder is distended with urine to determine whether UR is at the apex of the bladder; US can be repeated and performed with the bladder distended [15, 16]. Considering its simplicity and noninvasiveness, US is the best method for diagnosing UR. However, intestinal gas or abdominal wall thickness can prevent a detailed examination of the bladder site when using US. In such cases, MRI is an alternative option. CT is a useful tool, but it exposes patients to radiation, which is a disadvantage, especially for young children; therefore, its use should be limited as much as possible.

Our pathological examinations revealed that our surgical approach to UR allowed for complete UR excision. The results of preoperative imaging of the UR to determine its presence or absence were consistent with the pathological findings of UR. When UR ended before the bladder apex according to preoperative imaging, urachus tissue existed at the umbilicus but not at the fibromuscular cord-like structure according to intraoperative findings. These findings were also demonstrated pathologically. Therefore, the use of preoperative imaging was appropriate for determining the surgical margin and procedure. Our study proved that our surgical strategy presents good symptomatic UR treatment results with appropriate surgical margins and avoids unnecessary and excessive surgical invasion.

This study has several limitations. It was a single-center, retrospective case series. Our sample size was small; therefore, its results should be validated in a large population and at other institutions. Long-term follow-up was not performed; therefore, the potential risk of UR recurrence when complete excision was not achieved was not evaluated.

## Conclusions

When preoperative imaging revealed that UR continued toward the bladder apex, the pathological findings also indicated UR in the bladder margin. When preoperative imaging showed no UR near the bladder apex, the pathological findings indicated the UR near the umbilicus without continuing toward the bladder apex, and the TU approach was considered sufficiently appropriate for the complete excision of UR. When excision of the UR including the bladder apex is required, the TU approach should be initially selected, and if impossible, the LATU approach should be added. Our institution’s policy for determining the surgical approach based on preoperative imaging results is considered suitable for UR treatment in terms of complete UR resection and avoidance of excessive surgical invasion.

**Table 1:** Patients’ background and surgical outcomes (urachal sinus)

UR is limited at the umbilicus by preoperative imaging	
*Patients’ background*	
Age (year)*	6.5 (3-9.5)
Sex (male), n (%)	8 (80%)
*Preoperative imaging*	
Only at the umbilicus	5
Continue caudally from the umbilicus, but not continue toward the bladder apex	5
*Surgical outcomes*	
Approach	Trans-umbilical approach (10 cases)
Operation time (min)*	50 (40.5–64)
Hospitalization (postoperative day)*	1 (1–2)
Balloon (postoperative day)*	0 (0–0)
Complications, n (%)	0 (0)

UR, urachal remnant

*, median (interquartile range)

**Table 2:** UR continued toward the bladder apex by preoperative imaging

Approach	TU: 3 cases	LATU: 7 cases	*p* value
*Patients’ background*			
Age (year)*	1 (0.5–1.5)	11 (8.5–12)	0.02
Sex (male), n (%)	2 (67%)	5 (71%)	> 0.99
*Surgical outcomes*			
Operation time (min)*	59 (56–70)	131 (106.5–140.5)	0.02
Hospitalization (postoperative day)*	3 (3-3.5)	4 (3–4)	0.8
Balloon (postoperative day)*	3 (2-2.5)	2 (2–3)	0.7
Complications, n (%)	0 (0)	0 (0)	> 0.99

All cases were initially performed by TU approach (10 cases)

Three cases were completed by only TU approach, but seven cases required adding laparoscopic procedure, i.e. LATU approach was needed.

*UR* Urachal remnant;* TU* Trans-umbilical;* LATU* Laparoscopic-assisted trans-umbilical

*, Median (interquartile range)

Fisher’s exact test was used for discrete valuable. Wilcoxon Signed Rank test was used for continuous variable.

**Table 3:** Pathological diagnosis compared with preoperative imaging

	Preoperative UR imaging	
	Continued toward the bladder apex (n = 10)	Limited at near the umbilicus (n = 10)
*Pathological diagnosis*		
Urachal remnant	10	6
Umbilical polyp	0	1
Granulation tissue	0	1
Uncertain cases	0	2
*Resected margin*		
UR existed at the bladder site	Positive in 9 cases	None

*UR* Urachal remnant

## Data Availability

The datasets used and/or analyzed during the current study are available from the corresponding author upon reasonable request.

## Abbreviations

CT: Computed tomography
LATU: Laparoscopy-assisted trans-umbilicus
MRI: Magnetic resonance imaging
TU: Trans-umbilical
UR: Urachal remnant
US: Ultrasonography

## References

[1] Schubert GE, Pavkovic MB, Bethke-Bedürftig BA (1982). Tubular urachal remnants in adult bladders. J Urol.

[2] Gleason JM, Bowlin PR, Bagli DJ, Lorenzo AJ, Hassouna T, Koyle MA (2015). A comprehensive review of pediatric urachal anomalies and predictive analysis for adult urachal adenocarcinoma. J Urol.

[3] Liu Z, Yu X, Hu J, Li F, Wang S (2018). Umbilicus-sparing laparoscopic versus open approach for treating symptomatic urachal remnants in adults. Medicine (Baltim).

[4] Tanaka K, Misawa T, Baba Y, Ohashi S, Suwa K, Ashizuka S (2019). Surgical management of urachal remnants in children: open versus laparoscopic approach: a STROBE-compliant retrospective study. Medicine (Baltim).

[5] Fujiogi M, Michihata N, Matsui H, Fushimi K, Yasunaga H, Fujishiro J (2019). Early outcomes of laparoscopic versus open surgery for urachal remnant resection in children: a retrospective analysis using a nationwide inpatient database in Japan. J Laparoendosc Adv Surg Tech A.

[6] R Core Team (2021). R: a language and environment for statistical computing.

[7] Buddha S, Menias CO, Katabathina VS (2019). Imaging of urachal anomalies. Abdom Radiol (NY).

[8] Begg RC (1930). The urachus: its anatomy, histology and development. J Anat.

[9] Meeks JJ, Herr HW, Bernstein M, Al -Ahmadie Hikmat A, Dalbagni G (2013). Preoperative accuracy of diagnostic evaluation of the urachal mass. J Urol.

[10] Mylonas KS, O Malley P, Ziogas IA, El-Kabab L, Nasioudis D. Malignant urachal neoplasms: A population-based study and systematic review of literature. Urol Oncol. 2017;35:33.e11–33.e19. 10.1016/j.urolonc.2016.07.021 27592530

[11] Sato H, Furuta S, Tsuji S, Kawase H, Kitagawa H (2015). The current strategy for urachal remnants. Pediatr Surg Int.

[12] Nissen M, Rogge P, Sander V, Alrefai M, Romanova A, Tröbs R-B (2022). Pediatric urachal anomalies: monocentric experience and mini-review of literature. Child (Basel).

[13] Lipskar AM, Glick RD, Rosen NG, Layliev J, Hong AR, Dolgin SE (2010). Nonoperative management of symptomatic urachal anomalies. J Pediatr Surg.

[14] Neufang T (1992). Laparoscopic excision of an urahcal fistula: a new therapy for a rare disorder. Minim Invas Ther.

[15] Parada Villavicencio C, Adam SZ, Nikolaidis P, Yaghmai V, Miller FH (2016). Imaging of the urachus: anomalies, complications, and mimics. Radiographics.

[16] Yu JS, Kim KW, Lee HJ, Lee YJ, Yoon CS, Kim MJ (2001). Urachal remnant diseases: spectrum of CT and US findings. Radiographics.

